# DNA Damage after Continuous Irradiation: Findings in Mice Compared with Human Epidemiologic Data

**DOI:** 10.1289/ehp.1205564

**Published:** 2012-10-01

**Authors:** Jan Beyea

**Affiliations:** Consulting in the Public Interest, Lambertville, New Jersey, E-mail: jbeyea@cipi.com

Olipitz et al. (2012) suggested that their study of biomarkers in several hundred mice exposed to 10.5 cGy of ionizing radiation for 5 weeks casts into doubt radiation standards and concerns about protracted exposure after accidental releases of radioactivity. Yet, the authors failed to discuss the many human studies that have appeared in recent years showing excess cancers after protracted exposure (e.g., Cardis et al. 2005; Krestinina et al. 2007; Muirhead et al. 2009). The most likely explanation for the contradiction is that the biomarkers they examined are not predictive of cancer incidence 10–50 years after exposure, a possibility they did not mention. Before a cellular biomarker can be trusted to predict cancer risk, it first must be linked to epidemiologic data, something that Olipitz et al. have not done.

If Olipitz et al. (2012) interpreted their biomarker results correctly, then recent studies on humans must have been wrong. For example, in a study of 400,000 nuclear workers, Cardis et al. (2005) reported excess cancer from protracted exposure at a rate per Gray higher than that found in studies of one-time exposures in atomic bomb (A-bomb) survivors. In a study of 175,000 radiation workers receiving protracted exposures in the United Kingdom, Muirhead et al. (2009) observed excess cancer at the same rate as found in A-bomb survivors. Krestinina et al. (2007) found excess cancer in 17,000 members of the civilian population who received protracted exposure from emissions from the Soviet weapons complex—also at a higher rate than found in the A-bomb cohort. In addition, Chernobyl thyroid exposures meet the protracted test because > 90% of the dose came from iodine-131, which has an 8-day half-life (Gavrilin et al. 2004). It would have been helpful if Olipitz et al. (2012) had explicitly mentioned these epidemiologic contradictions to their data interpretation, thus allowing the reader to judge whether or not their mouse data should influence worker and public radiation standards for protracted exposures.

In the past, cellular radiation studies have conflicted with human epidemiologic data. Thus, the study by Olipitz et al. (2012) is not a test of the linear nonthreshold theory (LNT). The authors started with a dose almost universally accepted to cause a (small) risk of cancer if given all at once.

Perhaps Olipitz et al. (2012) would argue that the dose categories covered in the epidemiology studies cited above do not really include protracted exposures to 10.5-cGy doses, but only to doses no lower than 20 or 30 cGy. However, Olipitz et al. claimed to see “nothing” after 5 weeks, so the implication is that they would also see nothing after 10–15 weeks. If they thought otherwise, it would have been appropriate to say so. In addition, epidemiologic studies in regions with high natural background are not definitive. In one such study, Nair et al. (2009) concluded that their study in India, together with cancer mortality studies in China, could only set limits, suggesting that “it is unlikely that estimates of risk at low doses are substantially greater than currently believed.”

One of the biggest paradoxes in the debate on low-level radiation—whether about immediate or protracted exposure—is that an individual risk can be a minor concern, while the societal risk (the total delayed cancers in an exposed population) can be of major concern. Attempts to calm public overreaction should not ignore the human epidemiologic data. Further discussion of these controversies and their policy implications have been published previously (Beyea 2012).
